# Cardiorespiratory Fitness Predicted by Fibrinogen and Leptin Concentrations in Children with Obesity and Risk for Diabetes: A Cross-Sectional Study and a ROC Curve Analysis

**DOI:** 10.3390/nu13020674

**Published:** 2021-02-19

**Authors:** Kyriaki Tsiroukidou, Elpis Hatziagorou, Maria G. Grammatikopoulou, Anastasios Vamvakis, Kalliopi Kontouli, Christos Tzimos, John Tsanakas, Bessie E. Spiliotis

**Affiliations:** 1 3rd Department of Pediatrics, Hippokration General Hospital, 49 Konstantinoupoleos Str, Aristotle University of Thessaloniki, GR-54642 Thessaloniki, Greece; hatziagorou@auth.gr (E.H.); mariagram@auth.gr (M.G.G.); kkontoul@otenet.gr (K.K.); tsanakasj@gmail.com (J.T.); 2 Department of Nutritional Sciences & Dietetics, Faculty of Health Sciences, International Hellenic University, Alexander Campus, Sindos, P.O. Box 141, GR-57400 Thessaloniki, Greece; 3 Northern Greece Statistics Directorate, Hellenic Statistical Authority, GR-54646 Thessaloniki, Greece; ctzimos@gmail.com; 4 Paediatric Endocrine Research Laboratory, Division of Paediatric Endocrinology and Diabetes, Department of Paediatrics, School of Medicine, University of Patras, University Campus, GR-26504 Patras, Greece; besspil@endo.gr

**Keywords:** obese, overweight, childhood, cardiopulmonary test, insulin resistance, CPET, adiposity, VO_2max_, high-sensitive CRP, nutrition, exercise, metabolic health

## Abstract

Obesity is defined as abnormal or excessive fat accumulation that presents a risk to health. The ability to exercise is affected by adiposity, and this mechanism involves low-grade chronic inflammation and homeostatic stress produced mainly in adipocytes, which can result in abnormal adipokine secretion. To date, the gold standard for cardiorespiratory fitness assessment is considered to be the maximum oxygen uptake (VO_2max_). The aim of the present study was to assess the prognostic value of hematological parameters of childhood obesity, as potential predictors of cardiorespiratory fitness (VO_2max_), using a sample of children and adolescents with obesity and risk for diabetes. A total of 84 clinically healthy children and adolescents were recruited, of which 21 were considered lean, 22 overweight and 41 obese, with a mean age of 12.0 ± 1.9, 11.4 ± 2.0, and 11.2 ± 2.1 years old, in each weight status category, respectively. Age and sex did not differ between groups. Hematologic testing was performed after 12 h of fasting including glucose, serum lipids, insulin, hc-CRP, adiponectin, leptin and fibrinogen levels. Cardiorespiratory capacity for exercise was assessed to determine VO_2max_, using a cycle ergometer. The VO_2max_ was negatively correlated with progressive strength to the BMIz (−0.656, *p* ≤ 0.001), hs-CRP (r = −0.341, *p* ≤ 0.002), glucose (r = −0.404, *p* ≤ 0.001) and insulin levels (r = −0.348, *p* ≤ 0.001), the homeostasis model assessment of insulin resistance (HOMA-IR) (r = −0.345, *p* ≤ 0.002), as well as to the leptin (r = −0.639, *p* ≤ 0.001) and fibrinogen concentrations (r = −0.520, *p* ≤ 0.001). The multivariate analysis revealed that only leptin and fibrinogen concentrations could predict the VO_2max_ adjusted for the BMIz of participants. The receiver operating characteristic (ROC) curve for the diagnostic accuracy of leptin, hs-CRP and fibrinogen concentrations for the prediction of VO_2max_ revealed a good diagnostic ability for all parameters, with leptin being the most promising one (area under the curve (AUC): 99%). The results verify that in children with obesity, VO_2max_ may be predicted from hematological parameters (leptin and fibrinogen), possibly bypassing more invasive methods.

## 1. Introduction

Obesity is the result of a positive energy balance and/or physical inactivity, demonstrated by excessive body weight deposition for one’s height. The pathophysiology of obesity is complex and multifactorial, with the initial trigger stemming from homeostatic stress produced mainly in adipocytes, resulting in adipokine secretion attempting to balance energy homeostasis [1,2]. As a result, leptin levels are increased during obesity, with a parallel reduction in circulating adiponectin [3,4,5]. In parallel, variants of the adipokine-encoding genes are also affected [6], all adding to an increased risk for the development of diabetes.

Moreover, obesity is associated with a plethora of coagulation factors, all setting the basis for the establishment of the atherosclerotic process and cardiovascular disease (CVD) [7,8,9]. Of these, fibrinogen, a hemostatic protein produced by the hepatocytes, acts early on during the beginning of the atherothrombotic procedure [10,11]. Elevated fibrinogen concentrations have been reported in obese children and can serve as a prodromal atherosclerosis proxy index, for early intervention [8,12,13].

One of the most important results of obesity is the reduced cardiorespiratory fitness (CRF) observed due to exercise intolerance [14,15]. This phenomenon is in fact multifactorial, influenced by the hormonal background, the lack of motivation and engagement in physical activity (PA) compared to children with normal weight, as well as to the difficulty experienced in moving one’s body during exercise, further reducing CRF [14]. To date, the gold standard for CRF assessment is considered to be maximum oxygen uptake (VO_2max_) [14,16]. Overall, VO_2max_ is a difficult procedure for children, who often fail to achieve the reported standards [14,17,18], resulting in limited available data to date. On the other hand, VO_2max_ is influenced by both the genotype [19] and the phenotype, with obesity and insulin resistance being important CRF effectors [20]. In parallel, research has suggested that, through ameliorating CRF, we could attenuate the adverse events of obesity in children, by mediating the cardiometabolic risk and insulin resistance, hence delaying the progression to type 2 diabetes mellitus [21,22].

Studies have revealed a decreased CRF with increasing leptin and fibrinogen concentrations among children and adolescents [23,24,25,26,27] and a linear relationship between adiponectin and CRF [28]. With adipokines being influenced by physical activity [24] and vice versa [29], understanding their role is pivotal in exercise recommendations and primary and secondary prevention of obesity. Additionally, considering the difficulty in assessing VO_2max_ among children with obesity and risk for diabetes, it would be extremely useful if it could be predicted by more assessable indicators.

The aim of the present case-control study was to determine VO_2max_ levels among children of different weight status tiers and evaluate possible correlations between VO_2max_, serum leptin, adiponectin and fibrinogen levels.

## 2. Materials and Methods

### 2.1. Participant Recruitment and Inclusion Criteria

A total of 63 children and adolescents, 22 overweight and 41 with obesity (Table 1), aged 8–16 years old were recruited from the Endocrine outpatient Clinic of the 3rd Department of Pediatrics, of the Hippokration University Hospital, in Thessaloniki, Greece. Participants had scheduled appointments for overweight/obesity evaluation. Lean children and adolescents (*n* = 21) were recruited from local schools, via advertisements, forming the control group. Thus, the number of participants totaled 84 children and adolescents, who were divided into three groups, according to body weight status. Participant characteristics are presented in Table 1. Exclusion criteria included a diagnosis of (1) chronic diseases; (2) mental retardation; (3) failure to perform the cardiopulmonary exercise test; (4) young age of participants (below 8 years old); (5) posture difficulties affecting the ability to perform the CRF test; (6) inability to fulfill the prerequisite criteria for the VO_2max_ tests; (7) use of any medication.

### 2.2. Ethical Permission and Consent

Parents/guardians of participants signed informed consent forms prior to participation, after having the aims and method of the study explained in full detail. The protocol was approved by the Bioethics Committee of the Medical School of Aristotle University of Thessaloniki (Ref No. 91/21-12-2009). All data were handled with emphasis on anonymity and data protection, according to the Declaration of Helsinki and its latter amendments. This is a secondary analysis of a previous reported sample [26], with the use of a universally accepted weight status classification, inclusion criteria and participant categorization.

### 2.3. Anthropometric Indices

Body weight and height of participants were measured at approximately 9:00 a.m. by an experienced pediatrician (K.T.) and a dietitian (A.V.) using a Harpenden wall-mounted stadiometer (Holtain Ltd., Crymych, UK) and a Seca 700 mechanical column scale (Seca, Hamburg, Germany).

Body mass index (BMI) was calculated as the ratio of body mass (kg), towards the height of each participant, squared (m^2^). Weight status was defined according to the World Health Organization (WHO) international growth reference data for children and adolescents >5 years of age [30,31]. WHO Anthro software [32] was used to calculate the BMI z-scores (BMIz). Overweight and obesity were identified in children with a BMIz >1 standard deviation (SD) and BMIz >2SD, respectively [33].

Waist circumference was recorded using an anelastic measuring tape, with participants in the horizontal plane, midway between lower margin of the last palpable rib and the iliac crest [34], according to the WHO STEPS (STEPwise Approach to Surveillance) protocol [35]. Hips perimeter was measured at the widest portion of the buttocks [34], and waist/hip ratio was calculated accordingly.

### 2.4. Puberty Staging

Stage of sexual maturation was assessed by an experienced pediatric endocrinologist (K.T.), based on the Tanner stages criteria [36]. According to the sexual maturity rating, participants at stage 1 were considered as prepubertal, and all other stages were indicative of puberty.

### 2.5. Cardio-Pulmonary Exercise Test (CPET)

CRF was assessed with a cardio-pulmonary exercise test (CPET), performed on a cycle ergometer (Egromedic 828E, Monark Exercise, Vansbro, Sweden) using a spirometer (Sensormedics, Flowsensor, VMAX SERIES, v.20-1, Loma Linda, CA, USA) [14]. The test procedure was explained in detail to both the children and their guardians, and the first also had the opportunity to familiarize themselves with the cycle ergometer prior to the measurements.

The exercise protocol began with a resting period of 2 min for baseline measurements, followed by a 3 min warm-up period of unloaded cycling power. Exercise challenge consisted of progressively increasing increments of 10 Watt/min, followed by a recovery stage lasting for 3 min. All children were encouraged to exercise until the onset of exhaustion, throughout the test.

### 2.6. Blood and Biochemical Assays

Fasting venous blood was obtained from all participants at 9:00 a.m. and either assessed, or immediately centrifuged with the serum being stored at –80 °C. Stored serum samples were used for the analyses of triglycerides (TG) and high-density lipoprotein cholesterol (HDLC) levels, using Vitro Chemistry DT60 (Johnson & Johnson, NY, USA). Fasting blood glucose (FBG) levels were assessed with the hexokinase method (AU 2700 Analyzer, Beckman Coulter Inc., Orange County, CA, USA).

Citrated plasma samples were used for the assay of fibrinogen levels, using a commercially available kit (Multifriben U, SIEMENS), based on the Geftkin et al. [37] method.

Insulin levels were assessed using a Human Insulin ELISA kit (ALPCO Diagnostics) with both inter- and intra-assay precision being below 15%. The HOMA-IR (homeostasis model assessment of insulin resistance) was calculated by (fasting insulin (μU/mL) × fasting glucose (mM)/22.5) for insulin resistance [38].

Serum leptin and adiponectin levels were assessed with Human Leptin ELISA kits (Diagnostic Systems Laboratories, Texas, USA and AdipoGen, Seoul, Korea, respectively). The intra-assay coefficient of variation (CV) for adiponectin was 2.97–3.84% and the reported inter-assay ranging between 2.84 and 5.5%. The respective intra- and inter-assay CVs for leptin were 5.9 and 5.6%, respectively. High-sensitivity C-reactive protein (hs-CRP) was determined by a two-site chemiluminescent enzyme assay.

### 2.7. Statistical Analyses

All analyses were carried out with the Jamovi v. 1.2 (the Jamovi project) using the *R* language, and the SPSS statistical software v. 23.0 (IBM, Chicago, IL, USA). The level of significance was set at 5%. Initially, the general characteristics of variables were examined and, thereafter, we assessed all prerequisites for performing statistical analyses, including normality in distribution, variations, etc. Due to the uneven number of participants in each group, the bootstrap technique was applied for 10,000 identical items.

Differences between groups were assessed with ANOVA, using Bonferroni correction for normally distributed continuous variables. Such variables are presented as mean ± standard deviations (SD). For categorical variables, differences between groups were assessed with the Chi-square test. Correlations between variables were evaluated with the Pearson’s test.

Apart from crude parameters, the VO_2max_ was also adjusted to the BMIz of each participant and the calculated adjusted values were also used in the analyses.

For the assessment of the diagnostic ability of leptin, hs-CRP and fibrinogen in predicting VO_2max_, receiver operating characteristic (ROC) curves were also plotted [39].

Univariate and multivariate regression models were also designed, using quantitative variables. The effect of the degree of obesity in CRF was assessed with general linear models. Degree of obesity was converted to a dummy variable for use in multiple regression models, in order to better define the best model explaining the results, using stepwise and enter methods. Residual distribution was assessed in all cases, using the Kolmogorov–Smirnov test. Models were also assessed for tolerance and collinearity.

## 3. Results

### 3.1. Differences in Anthropometry, Hormonal Levels and CRF

Table 2 details between-group differences in the anthropometric, exercise and biochemical characteristics of participants. As expected, anthropometric indices were different between groups, with obese participants demonstrating greater BMI, BMIz, waist and hips circumferences, as well as waist/hips ratio. HOMA-IR and leptin levels were significantly lower among lean controls, compared to the obese, but in contrast, VO_2max_ was significantly higher. Overall, VO_2max_ was significantly reduced at each increasing obesity tier. In parallel, leptin and fibrinogen levels were significantly increased at each higher adiposity level. The results remained similar for all of the aforementioned parameters, even after dividing the sample according to sex, with the exception of fibrinogen levels. 

In Table 3, differences in the anthropometric, CRF and biochemical characteristics of participants are presented according to puberty and weight status. With regard to the anthropometric indices, all were significantly increased with each ascending weight status tier, in both prepubertal and pubertal children. Irrespective of puberty status, VO_2max_ was decreased in each ascending adiposity level, whereas circulating leptin and fibrinogen levels were increased, in parallel to the weight status.

### 3.2. Correlations between VO_2max_, Anthropometric Parameters and Hormonal Levels

In the total sample, the crude VO_2max_ (expressed as mL/Kg/min) demonstrated negative correlations of progressive strength to the BMIz (−0.656, *p* ≤ 0.001), hs-CRP (r = −0.341, *p* ≤ 0.002), glucose (r = −0.404, *p* ≤ 0.001) and insulin levels (r = −0.348, *p* ≤ 0.001), HOMA-IR (r = −0.345, *p* ≤ 0.002), leptin (r = −0.639, *p* ≤ 0.001) and fibrinogen levels (r = −0.520, *p* ≤ 0.001) of participating children. On the other hand, a weak, positive correlation was observed with adiponectin (r = 0.329, *p* ≤ 0.003).

When the VO_2max_ was expressed as a percent (%), all aforementioned correlations either remained similar, or were strengthened, becoming more significant, with the exception of adiponectin (r = 0.243, *p* ≤ 0.05). In more detail, the VO_2max_ (%) was negatively associated with fibrinogen levels (r = −0.530, *p* ≤ 0.001), leptin (r = −0.624, *p* ≤ 0.001), glucose (r = −0.365, *p* ≤ 0.001) and insulin levels (r = −0.419, *p* ≤ 0.001), HOMA-IR (r = −0.428, *p* ≤ 0.002), hs-CRP (r = −0.380, *p* ≤ 0.001), and BMIz (−0.772, *p* ≤ 0.001).

### 3.3. Univariate and Multivariate Regression Models Predicting VO_2max_

Linear correlations with VO_2max_ adjusted for BMIz and the multivariate regression models predicting VO_2max_ (adjusted) by biochemical parameters, are presented in Table 4. In the univariate analysis, all variables demonstrated significant correlations with the adjusted VO_2max_.

According to the multivariate analysis, only leptin and fibrinogen levels could predict the VO_2max_. The adjusted R^2^ of the multivariate regression model was equal to 0.527 and although the correlation between leptin and fibrinogen as independent variables was rather strong (r = −0.552, *p* < 0.001), no collinearity issues existed, as the portion of variance that was not explained by the other variable (tolerance) was greater than 0.73. In addition, the remaining multicollinearity indicators, including the variance inflation factor (VIF), Eigen values, the condition index and variance proportions, were within the accepted thresholds.

### 3.4. Diagnostic Accuracy of Leptin, hs-CRP and Fibrinogen for Predicting VO_2max_

**Figure 1:**
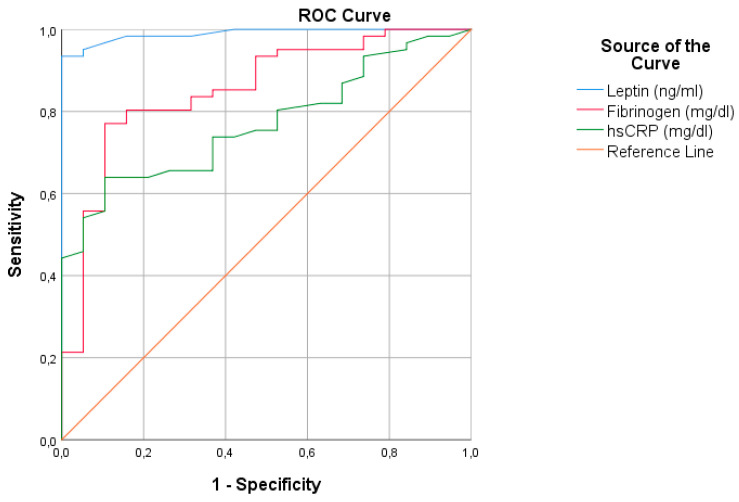
ROC curve of threshold leptin, hs-CRP and fibrinogen levels for predicting VO_2max_ (sensitivity versus specificity). *hsCRP*, high sensitivity C-reactive protein; *ROC*, receiver operating characteristic; VO_2max_, maximal aerobic capacity.

The ROC curve for the diagnostic accuracy of leptin, hs-CRP and fibrinogen concentrations for the prediction of VO_2max_ is presented in Figure 1. All parameters had a good diagnostic ability, with leptin being the most promising one. The area under the curve (AUC) of leptin was 99% with the respective 95% confidence intervals (CI) between 97.4 and 100%. The AUC of fibrinogen was 85.3% (95% CI: 75.5–95.2%) and that of hs-CRP was estimated at 77.0% (95% CI: 66.7–87.3%).

## 4. Discussion

Our study revealed that in children with overweight and obesity, leptin and fibrinogen concentrations are strongly correlated to the VO_2max_ and may predict the latter.

Adipokines and insulin have been shown to interplay with CRF [29]. According to a recent study [40], adolescents diagnosed with obesity and type 2 diabetes (T2DM) demonstrated significantly reduced CRF compared to those without T2DM. Additionally, Agostinis-Sobrinho and associates [28] observed an inverse association between serum adiponectin levels and CRF, among lean, but not overweight adolescents. It is well known that adiponectin has anti-atherogenic and anti-inflammatory properties [41], whereas on the other hand, leptin safeguards energy homeostasis by promoting weight loss and increasing energy output [42]. Subsequently, in the present study, as VO_2max_ decreased among overweight children, an increment in leptin levels and a parallel decrease in circulating adiponectin concentrations were observed, triggering the production of more energy.

In accordance with the literature, the present, secondary analysis of previous data verified the existence of associations concerning leptin, adiponectin and hs-CRP with CRF, irrespective of the weight status categorization applied. Studies to date have shown that reduced CRF is associated with increased leptin [23,24,25] and reduced adiponectin [26,28] levels among children and adolescents. As far as hs-CRP is concerned, studies have verified the existence of a negative association between CRF and hsCRP as a marker of inflammation [43,44], CVD risk [45], and compromised liver function [46]. Additionally, hs-CRP has also been proposed as a prognostic factor of CRF among adults with systemic inflammation [44]. As far as dietary manipulations are concerned, randomized controlled trials (RCTs) have revealed that hs-CRP levels are reduced following a systematic moderate red wine intake [47], when adopting a Mediterranean dietary pattern [48], or energy restrictive diets, irrespective of their macronutrient composition [49,50]. On the other hand, based on observational data, higher hs-CRP concentrations are associated with lower diet quality and lower adherence to the Mediterranean diet [51]. 

With regard to the fibrinogen concentrations, in the present analysis it was shown that they have a good diagnostic ability for the prediction of VO_2max_. Lewitt and Baker [27] also revealed a negative relationship between fibrinogen levels and CRF among children and adolescents, indicating the initiation of a hypercoagulable state [52]. In parallel, fibrinogen has also been associated with hs-CRP in children with overweight and obesity [12]. In a follow-up study of approximately 2000 children [44], increased childhood fitness was associated with lower inflammation during the adult life and reduced fibrinogen in men. Thus, it appears that the interplay of CRF and fibrinogen during childhood might be an effector of adult CRF and coagulation status. Nutrition-wise, apart from obesity and increased body fat [53], observational studies have also associated elevated fibrinogen concentrations to greater iron, sugar, and caffeine intake [54], lower fiber content of the diet [55] and consumption of less than 1 kiwi weekly [56]. In parallel, clinical trials have revealed a reduction in fibrinogen levels following dietary protein restriction [57]. On the other hand, animal studies have revealed an elevation of fibrinogen levels, and the development of fibrin(ogen) deposits in the liver and white adipose tissue in mice, following a high-fat diet [58].

Taken et al. [59] showed that the pediatric exercise testing is imperative in assessing CVD risk. Moreover, according to a recent meta-analysis of longitudinal studies [60], early life CRF is associated with inverse outcomes concerning adult BMI, adiposity and metabolic syndrome. Obese children often demonstrate sleep apnea, dyspnea, several respiratory limitations and lung function abnormalities, leading to exercise intolerance [61]. CRF is an important contributor to the overall health, respiratory function and weight status of children. As reduced CRF is observed in children with obesity compared to their leaner counterparts [59], lung function is also compromised. According to the MINISTOP (Mobile-based INtervention Intended to STop Obesity in Preschoolers) trial [62], elevated body fat is associated with worse CRF, lower-body muscular strength and motor fitness among pre-schoolers. However, according to the literature [63,64,65,66], reduced CRF is due to impaired lung function only among children with increased adiposity, as a result of lung compliance. With CRF assessment being a difficult task in overweight children and in children with obesity, the use of easier methods predicting it in children is crucial. As such, simple assays including leptin, fibrinogen and hs-CRP levels might be able substitutes for the burdensome test performed on a cycle ergometer and may provide reliable data concerning CRF to the pediatric obesity management team. In Greece, during the previous decade, obesity rates have been soaring [67,68,69,70], with overweight children of increased severity demonstrating reduced physical activity levels [71]. According to Thivel and Aucouturier [14], assessment of CRF is an important clinical parameter for evaluating the functional health of children and is necessary during the implementation of obesity treatment interventions, in particular the ones based on physical activity. Thus, turning the spotlight on CRF assessment in pediatric obesity management, using assessments that are less difficult to perform, is important for proactive weight management.

Table 5 details the available evidence on the possible role of nutrition in improving CRF among children with obesity, provided by RCTs. Overall, the evidence suggest that exercise interventions appear superior to nutrition alone in improving CRF and reversing progress to diabetes by improving insulin sensitivity among children and adolescents with overweight/obesity. A long-term exercise program (6 months) is also efficient in reducing biomarkers of inflammation and improving endothelial dysfunction among adolescents with obesity [72]. In parallel, a lack of studies assessing nutrition interventions alone, without exercise, is apparent. For this, although nutrition is a known effector for the prevention of diabetes and obesity [73], with regard to the CRF, the need for incorporating exercise in the interventions is highlighted from the available primary evidence (Table 5) and recent meta-analyses [74].

Nevertheless, the CRF assessment entails several restrictions in daily clinical practice. Personnel with expertise is required, as well as suitable equipment. As a result, few pediatric clinics have units equipped for assessing the CRF. Children and adolescents with obesity often find it difficult to respond to the CPET as a result of emotional, psychological and behavioral disorders associated with obesity [79], complimented by the fact that they tend to avoid any kind of exercise. With the aid of hematological parameters and by taking the degree of obesity into consideration, exercise ability can be more easily defined [14].

A strong point in the present study was the favorable response of participants to the CPET, using the cycle ergometer. This could be due to the study design (informing the family, experienced personnel) but also to the method selection for the CRF assessment. In children, CPET using the cycle ergometer seems to outweigh that performed on the treadmill. Furthermore, an additional strong point in our study was the application of the CPET for assessment of the exercise ability and not only for defining the VO_2max_.

Limitations of the present study include the relatively small sample used and the cross-sectional design and indirect definition of the body fat. BMI was selected as the most widely accepted method for indirect body fat assessment in children and adolescents. However, important aspects of the study herein include the assessment of VO_2max_ in a pediatric population, and its prediction by easy-to-perform assays.

Intervention studies have showed that diet and exercise consist of cost-effective strategies in improving metabolic profile among children with obesity, as well as among those with concomitant respiratory issues, and, as such, they could be employed to tamper the problem and mitigate the prevalence of type 2 diabetes mellitus in the young [80,81,82,83]. More research is required to assess the effect of nutrition interventions in improving CRF among children with overweight or obesity.

## 5. Conclusions

Childhood obesity is accompanied by too many comorbidities [84,85,86]. The present study verifies previous analyses [26] suggesting that in obese children, VO_2max_ can be predicted by hematological parameters (leptin and fibrinogen), bypassing more invasive methods. Early metabolic disorders due to childhood and adolescent obesity seem to affect cardiovascular fitness for exercise and their timely assessment is mandatory in order to protect children and adolescents with obesity from metabolic and cardiovascular complications, and reducing the risk for developing diabetes.

## Figures and Tables

**Table 1:** Participant characteristics (mean ± SD or *n*).

Age (years)	11.5 ± 2.0
Sex (boys/girls) (*n*)	42/42
Prepubertal/pubertal (*n*)	26/58
BMIz	2.26 ± 1.46
Weight status (normoweight/overweight/obese) (*n*)	21/22/41

BMIz: body mass index z-score [30,31]; SD: standard deviation.

**Table 2:** Anthropometric, biochemical and exercise test parameters of participants according to sex and weight status (mean ± SD).

Characteristics	Normoweight			Overweight			Obese			*p*-Value between Weight Status Tiers
	Boys (*n* = 9)	Girls (*n* = 12)	Total (*n* = 21)	Boys (*n* = 9)	Girls (*n* = 13)	Total (*n* = 22)	Boys (*n* = 24)	Girls (*n* = 17)	Total (*n* = 41)	
Age (years)	12.3 ± 2.2	11.9 ± 1.7	12.0 ± 1.9	12.0 ± 1.8	10.9 ± 2.1	11.4 ± 2.0	11.3 ± 2.3	11.1 ± 1.9	11.2 ± 2.1	NS
Prepubertal/pubertal (*n*)	4/5	1/11	5/16	2/7	3/10	5/17	13/11	3/14	16/25	NS
Body weight (kg)	45.2 ± 13.9	41.2 ± 11.6 ^‡^	42.8 ± 12.3 ^‡‡‡^	57.7 ± 13.3	55.9 ± 9.9 *	56.6 ± 11.2 ^‡‡^*	71.2 ± 24.3 *	69.9 ± 14.0 ***	70.6 ± 20.5	<0.001
Height (m)	1.53 ± 0.16	1.48 ± 0.13	1.50 ± 0.14	1.55 ± 0.17	1.49 ± 0.1	1.52 ± 0.13	1.5 ± 0.12	1.5 ± 0.1	1.51 ± 0.11	ΝS
BMI (kg/m^2^)	18.8 ± 2.4 ^‡‡‡^	18.3 ± 2.6 ^‡‡‡^	18.5 ± 2.5 ^‡‡‡^	23.7 ± 1.0 **	24.9 ± 1.7 ***	24.4 ± 1.6 ^‡‡‡^***	30.0 ± 3.2 ***	29.6 ± 3.3 ***	29.8 ± 3.22	<0.001
BMIz	0.4 ± 0.62 ^‡‡‡^	0.03 ± 1.02 ^‡^	0.18 ± 0.88 ^‡‡‡^	2.03 ± 0.48 ***	2.24 ± 0.4 ***	2.15 ± 0.43 ^‡‡‡^***	3.57 ± 0.77 ***	2.98 ± 0.56 ***	3.33 ± 0.74	<0.001
Waist circumference (cm)	64 ± 9.7 ^‡^	64.4 ± 8.1 ^‡^	64.2 ± 8.6 ^‡‡‡^	84.6 ± 4.7 *	86.6 ± 9.3 ***	85.8 ± 7.7 ^‡‡‡^***	100.3 ± 17.7 ***	100.4 ± 12.4 ***	100.33 ± 15.53	<0.001
Hips circumference (cm)	78.1 ± 10.3	81.0 ± 9.3 ^‡^	79.7 ± 9.6 ^‡‡‡^	91.3 ± 8.5	94.3 ± 8.9 **	93.1 ± 8.6 ^‡‡‡^ **	101.3 ± 13.9 ***	103.1 ± 7.8 ***	102.05 ± 11.67	<0.001
Waist/hips ratio	0.82 ± 0.02	0.8 ± 0.05	0.81 ± 0.04 ^‡‡‡^	0.93 ± 0.06 **	0.92 ± 0.04 ***	0.92 ± 0.05 ^‡‡‡^**	0.99 ± 0.08 ***	0.97 ± 0.08 ***	0.98 ± 0.08	<0.001
hs-CRP (mg/dL)	0.13 ± 0.09	0.14 ± 0.09	0.13 ± 0.09	0.23 ± 0.16	0.28 ± 0.27	0.26 ± 0.23	0.78 ± 1.32	0.39 ± 0.33	0.62 ± 1.04	0.040
Glucose (mg/dL)	75.7 ± 7.0	82.4 ± 6.4	79.5 ± 7.3 ^‡‡‡^	84.2 ± 7.5	84.3 ± 8.1	84.3 ± 7.6	88.5 ± 7.8 ***	87.5 ± 6.0	88.1 ± 7.03	<0.001
Insulin (μUI/mL)	2.6 ± 1.5	3.1 ± 2.4	2.9 ± 2.1 ^‡‡‡^	5.6 ± 4.8	7.4 ± 5.1	6.6 ± 4.9	13.5 ± 17.1	12.4 ± 8.0 ***	13.07 ± 13.93	0.001
TG/HDL	0.9 ± 0.5	1.3 ± 0.5	1.1 ± 0.5 ^‡‡^	1.8 ± 1.1	2.1 ± 1.3	1.9 ± 1.2	2.0 ± 1.3 *	2.3 ± 1.4	2.13 ± 1.31	0.006
HOMA-IR	0.5 ± 0.3	0.6 ± 0.5	0.6 ± 0.4 ^‡‡^	1.2 ± 1.2	1.6 ± 1.2	1.4 ± 1.2	3.1 ± 4.2	2.7 ± 1.7 ***	2.92 ± 3.34	0.001
VO_2max_ (mL/kg/min)	48.3 ± 6.9 ^‡^	46.4 ± 7.6	48.7 ± 6.2 ^‡‡‡^	38.1 ± 6.7 **	33.6 ± 5.0 ***	35.7 ± 6.0 ***^‡‡‡^	31.5 ± 5.9 ***	28.4 ± 5.3 ***	30.38 ± 5.95	<0.001
VO_2max_ (%)	95.7 ± 8.2 ^‡^	107.5 ± 15.4	102.9 ± 14.1 ^‡‡‡^	74.1 ± 14.0 **	74.9 ± 11.1 ***	75.1 ± 12.1 ***^‡‡‡^	58.5 ± 11.1 ***	65 ± 11.8 ***	61.32 ± 11.74	<0.001
Leptin (ng/mL)	4.0 ± 3.2 ^‡^	6.5 ± 3.5	5.5 ± 3.5 ^‡‡‡^	21.9 ± 12.7 *	30 ± 9.3 ***	25.5 ± 11.8 ***^‡‡‡^	37.1 ± 16.0 ***	42.5 ± 20.7 ***	39.65 ± 18.98	<0.001
Adiponectin (μg/mL)	15.8 ± 6.5	14.9 ± 6.8	15.3 ± 6.5	14.5 ± 5.2	14.9 ± 9.2	14.7 ± 7.5	13.1 ± 5.7	10.5 ± 3.9	12.06 ± 5.15	NS
Fibrinogen (mg/dL)	235.6 ± 68.8	253.5 ± 69.3	245.0 ± 67.8 ^‡‡‡^	324.2 ± 61.7 *	302.4 ± 87.9	311.0 ± 77.4 *^‡‡^	373.4 ± 61.2 ***	384.4 ± 99.5 ***	378.0 ± 78.4	0.001

BMI: body mass index; BMIz: BMI z-score [30,31]; HDL: high-density lipoprotein; HOMA-IR: homeostatic model assessment for insulin resistance; hs-CRP: high-sensitivity C-reactive protein; NS: not significant; SD: standard deviation; TG: triglycerides; VO_2max_: maximal aerobic capacity; ^‡^ Statistically different compared to the children of the same sex, with obesity, based on the post-hoc analyses (^‡‡‡^ *p* ≤ 0.001, ^‡‡^ *p* ≤ 0.01, ^‡^ *p* < 0.05); * statistically different compared to the normoweight children of the same sex, based on post-hoc analyses (*** *p* ≤ 0.001, ** *p* ≤ 0.01, * *p* < 0.05).

**Table 3:** Anthropometric, biochemical and exercise test parameters of participants according to puberty stage and weight status (mean ± SD).

	Prepubertal			Pubertal		
Characteristics	Normoweight (*n* = 5)	Overweight (*n* = 5)	Obese (*n* = 16)	Normoweight (*n* = 16)	Overweight (*n* = 17)	Obese (*n* = 25)
Age (years)	10.3 ± 1.0	10.0 ± 1.4	9.6 ± 1.3	12.6 ± 1.8	11.8 ± 2.0	12.2 ± 1.9
Boys/girls (*n*)	4/1	2/3	13/3	5/11	7/10	11/14
Body weight (kg)	33.8 ± 5.0	46.5 ± 5.7 ^‡^	58.3 ± 9.1 ***	45.8 ± 12.7	59.6 ± 10.7 ^‡‡^	78.5 ± 21.9 ***
Height (m)	1.4 ± 0.07	1.39 ± 0.1	1.42 ± 0.06	1.54 ± 0.14	1.55 ± 0.13	1.57 ± 0.1
BMI (kg/m^2^)	17.1 ± 1.5	24.1 ± 1.4 ***^‡‡^	28.8 ± 2.7 ***	19.0 ± 2.6	24.5 ± 1.6 ***^‡‡‡^	30.5 ± 3.4 ***
BMIz	0.18 ± 0.59	2.41 ± 0.14 ***^‡^	3.74 ± 0.71 ***	0.18 ± 0.98	2.08 ± 0.46 ***^‡‡‡^	3.06 ± 0.65 ***
Waist circumference (cm)	57.0 ± 5.3	81.3 ± 6.8 **	93.7 ± 12.3 ***	67.0 ± 8.0	87.1 ± 7.6 ***^‡‡‡^	104.6 ± 16.1 ***
Hips circumference (cm)	70.2 ± 4.6	85.9 ± 5.8 *	94.6 ± 8.7 ***	83.4 ± 8.5	95.2 ± 8.3 **^‡‡‡^	106.8 ± 11.0 ***
Waist/hips ratio	0.81 ± 0.02	0.95 ± 0.04 **	0.99 ± 0.07 ***	0.8 ± 0.05	0.9 ± 0.1 ***^‡^	0.98 ± 0.08 ***
hs-CRP (mg/dL)	0.18 ± 0.12	0.31 ± 0.24	0.69 ± 1.01	0.12 ± 0.08	0.24 ± 0.23	0.58 ± 1.08
Glucose (mg/dL)	76.6 ± 7.4	85.0 ± 4.6	86.7 ± 6.0 *	80.4 ± 7.3	84.1 ± 8.4	89.0 ± 7.57 **
Insulin (μUI/mL)	2.0 ± 0.0	4.1 ± 4.0	13.1 ± 19.3	3.1 ± 2.3	7.2 ± 5.0 ^‡^	13.1 ± 9.6 ***
TG/HDL	0.7 ± 0.3	2.5 ± 1.5	1.8 ± 1.5	1.3 ± 0.5	1.8 ± 1.1	2.3 ± 1.2 **
HOMA-IR	0.38 ± 0.04	0.86 ± 0.89	2.85 ± 4.42	0.63 ± 0.48	1.58 ± 1.22	2.97 ± 2.52
VO_2max_ (mL/kg/min)	46.7 ± 8.4	35.9 ± 8.2 *	32.2 ± 6.9 **	47.3 ± 7.1	35.3 ± 5.6 ***^‡‡^	29.0 ± 4.8 ***
VO_2max_ (%)	102.7 ± 21.9	73.4 ± 10.9	60.7 ± 13.5 ***	102.9 ± 13.1	74.9 ± 12.7 ***^‡‡^	61.7 ± 10.83 ***
Leptin (ng/mL)	4.1 ± 3.2	27.8 ± 8.6	38.9 ± 20.3 **	5.9 ± 3.6	26.4 ± 12.2 ***^‡‡^	39.6 ± 17.0 ***
Adiponectin (μg/mL)	18.6 ± 4.0	18.0 ± 4.2	13.7 ± 4.1	14.3 ± 6.9	14.4 ± 7.8	11.0 ± 5.6
Fibrinogen (mg/dL)	244.2 ± 64.4	263.6 ± 42.2 ^‡‡^	367.6 ± 53.9 ***	245.9 ± 71.1	325.3 ± 80.6 *	384.6 ± 91.1 ***

BMI: body mass index; BMIz: BMI z-score [30,31]; HDL: high-density lipoprotein; HOMA-IR: homeostatic model assessment for insulin resistance; hs-CRP: high-sensitivity C-reactive protein; NS: not significant; SD: standard deviation; TG: triglycerides; VO_2max_: maximal aerobic capacity; ^‡^ statistically different compared to the children of the puberty category, with obesity, based on the post-hoc analyses (^‡‡‡^ *p* ≤ 0.001, ^‡‡^ *p* ≤ 0.01, ^‡^ *p* < 0.05); * statistically different compared to the normoweight children of the puberty category, based on post-hoc analyses ( *** *p* ≤ 0.001, ** *p* ≤ 0.01, * *p* < 0.05).

**Table 4:** Linear regression model explaining cardiorespiratory fitness (VO_2max_) adjusted for the level of adiposity (BMIz) and biochemical parameters.

	Univariate Analysis			Multivariate Analysis		
Variables:	Unadjusted β	95% CI	*p*-Value	Adjusted β	95% CI	*p*-Value
Leptin (ng/mL)	0.601	(−0.739 to −0.463)	<0.001	0.492	(−0.661 to −0.323)	<0.001
Fibrinogen (mg/dL)	0.092	(−0.126 to −0.059)	<0.001	0038	(−0.074 to −0.002)	0.038
Adiponectin (μg/dL)	0.665	(0.075 to 1.255)	0.028	0.211	(−0.302 to 0.725)	NS
hs-CRP (mg/dL)	5.938	(−10.419 to −1.457)	0.010	0.691	(−4.889 to 3.508)	NS

BMIz, body mass index z-score [30,31]; CI, confidence intervals; hs-CRP, high-sensitivity C-reactive protein; NS, not significant; VO_2max_, maximal aerobic capacity.

**Table 5:** Existing parallel RCTs assessing exercise and nutrition interventions for improving CRF among children and adolescents with overweight/obesity.

First Author	Study Origin	Participants	Intervention(s)	Comparator	Intervention Duration	Results
Plavsic [75]	Serbia	Adolescent girls with obesity (N = 44, aged 13–19 years)	Dietary advice on CV biomarkers, hormonal parameters, and cardiorespiratory fitness and HIIT (*n* = 22)	Dietary advice only (*n* = 22)	12 weeks	The intervention group increased insulin sensitivity index and workload and decreased glucose AUC, insulin AUC, and hs-CRP compared with the diet group.
Ingul [76,77]	Norway	Children with obesity (N = 99)	(1) HIIT (*n* = 33), including 4 × 4 min bouts at 85–95% HR_max_ 3 times/week and nutrition advice (2) MICS (*n* = 32), 44 min at 60–70% HR_max_, 3 times/week and nutrition advice	Nutrition advice only (*n* = 34)	12 weeks	HIIT and MICS were equally efficacious and superior to nutrition alone, for improving LVS. HIIT was effective in increasing CRF when compared with the MICS and nutrition interventions.
Seo [78]	Korea	Children and adolescent with moderate to severe obesity(N = 103)	Exercise intervention (*n* = 32)	Usual care (including nutrition and exercise advice) (*n* = 71)	16 weeks	Only the exercise group had a significantly lower BMIz compared to the baseline. Significant group by time interaction was observed in %BF, LBM, DBP, hs-CRP and wall-sit test.

%BF, percent body fat; AUC, area under the curve; BMI, body mass index; BMIz, body mass index z-score; CRF, cardiorespiratory fitness; CV, cardiovascular; DBP, diastolic blood pressure; HIIT, high-intensity interval training; hs-CRP, high-sensitivity C-reactive protein; HR_max_, maximum heart rate; LBM, lean body mass; LVS, left ventricular peak systolic tissue velocity; MICS, moderate intensity continuous training; PWV, pulse wave velocity; RCT, randomized controlled trial; RT, resistance training.

## Data Availability

Data are available upon request by the study’s statistician (C.T.).
